# A novel, robust peptidyl-lys metalloendopeptidase from *Trametes coccinea* recombinantly expressed in *Komagataella phaffii*

**DOI:** 10.1007/s00253-023-12986-3

**Published:** 2024-01-13

**Authors:** Uzair Ahmed, Tobias Stadelmann, Daniel Heid, Berit Würtz, Jens Pfannstiel, Katrin Ochsenreither, Thomas Eisele

**Affiliations:** 1 Faculty of Mechanical and Process Engineering, Hochschule Offenburg, 77652 Offenburg, Germany; 2 Department of Chemical and Process Engineering, Karlsruhe Institute of Technology (KIT), 76131 Karlsruhe, Germany; 3 Mass Spectrometry Unit Core Facility, University of Hohenheim, 70599 Stuttgart, Germany

**Keywords:** LysN, Acidic endopeptidase, Disulfide mapping, Trypsin, Proteomics, Kex2, Zymogen, Maturation, Peptidyl-lys metalloendopeptidase

## Abstract

**Abstract:**

A novel peptidyl-lys metalloendopeptidase (*Tc*-LysN) from *Tramates coccinea* was recombinantly expressed in *Komagataella phaffii* using the native pro-protein sequence. The peptidase was secreted into the culture broth as zymogen (~38 kDa) and mature enzyme (~19.8 kDa) simultaneously. The mature *Tc*-LysN was purified to homogeneity with a single step anion-exchange chromatography at pH 7.2. N-terminal sequencing using TMTpro Zero and mass spectrometry of the mature *Tc-*LysN indicated that the pro-peptide was cleaved between the amino acid positions 184 and 185 at the Kex2 cleavage site present in the native pro-protein sequence. The pH optimum of *Tc*-LysN was determined to be 5.0 while it maintained ≥60% activity between pH values 4.5—7.5 and ≥30% activity between pH values 8.5—10.0, indicating its broad applicability. The temperature maximum of *Tc*-LysN was determined to be 60 °C. After 18 h of incubation at 80 °C, *Tc*-LysN still retained ~20% activity. Organic solvents such as methanol and acetonitrile, at concentrations as high as 40% (v/v), were found to enhance *Tc*-LysN’s activity up to ~100% and ~50%, respectively. *Tc*-LysN’s thermostability, ability to withstand up to 8 M urea, tolerance to high concentrations of organic solvents, and an acidic pH optimum make it a viable candidate to be employed in proteomics workflows in which alkaline conditions might pose a challenge. The nano-LC-MS/MS analysis revealed bovine serum albumin (BSA)’s sequence coverage of 84% using *Tc*-LysN which was comparable to the sequence coverage of 90% by trypsin peptides.

**Key points:**

•*A novel LysN from Trametes coccinea (Tc-LysN) was expressed in Komagataella phaffii and purified to homogeneity*

•*Tc-LysN is thermostable, applicable over a broad pH range, and tolerates high concentrations of denaturants*

•*Tc-LysN was successfully applied for protein digestion and mass spectrometry fingerprinting*

**Supplementary Information:**

The online version contains supplementary material available at 10.1007/s00253-023-12986-3.

## Introduction

Peptidyl-lys metalloendopeptidases, more commonly known as LysN (EC 3.4.24.20), are enzymes that preferentially hydrolyze substrates at the N-terminus of the lysine residues (Barrett et al. 2004). The first LysN peptidases were isolated from various bacterial and fungal sources, such as *My*-LysN (from *Myxobacter* AL-1), *Am*-LysN (from *Armillaria mellea*), *Po-*LysN (from *Pleurotus ostreatus)*, and *Gf-*LysN (from *Grifola frondosa)* (Dohmae et al. 1995; Lewis et al. 1978; Nonaka et al. 1995; Wingard et al. 1972). These early reports concluded that peptidases in this group have a pH optimum that ranges from neutral to alkaline (pH 7.0–9.5)*.* These enzymes were also found to be quite thermostable and exhibited relatively high resistance to denaturants such as urea and guanidine hydrochloride. *Gf*-LysN (Saito et al. 2002) and *Am*-LysN (Ødum et al. 2016) have also been recombinantly expressed in *Komagataella phaffii*, both as inactive protein (zymogen) and mature (active) protein using the full-length native pre-pro-protein, pro-protein, and mature protein coding sequences.

As a self-protection mechanism against inadvertent proteolysis within the cell, endopeptidases are often recombinantly expressed and secreted as inactive precursors, called zymogens or proenzymes, containing inhibitory N-terminal pro-peptides (Demidyuk et al. 2010). The cleavage of pro-peptides from these inactive precursor proteins sometimes occurs through proteolysis by pro-protein convertases within the host’s secretory pathway, resulting in the production of biologically active proteins or peptides. Kex2, also known as kexin (EC 3.4.21.61), was the first identified pro-protein convertase involved in the processing of α-mating factor and killer toxin precursors in *Saccharomyces cerevisiae.* Kex2 preferentially hydrolyzes peptide bonds at the C-terminus of lysine-arginine and arginine-arginine residues (Fuller et al. 1988). Proper protein folding and secretion are quite often the two limiting steps in heterologous protein expression. Even small changes in the pro-peptide sequence can have a consequential impact on the expression and activity of the recombinant peptidase (Boon et al. 2020). Higher expression levels of recombinant *Am*-LysN were reported by Ødum et al. (2016) when native pro-peptide sequence was used.

Owing to their strict cleavage specificity, high thermostability, and ability to withstand denaturants, LysN peptidases—*Gf*-LysN, especially—have attracted researchers to explore the potential of their application in proteomics experiments. *Gf*-LysN has been reported to perform equally well as trypsin, which is the preferred peptidase for mass spectrometry (MS)-based proteomics (Taouatas et al. 2010). Trypsin, however, may fail to produce MS-identifiable peptides derived from the carboxy termini of proteins due to the lack of amino acids that can easily accept protons. To better identify C-terminal peptides, peptidases that cleave at the N-terminal of basic amino acids such as LysN (Raijmakers et al. 2010; Taouatas et al. 2008) and the recently introduced LysargiNase (Huesgen et al. 2014; Tallant et al. 2006) could be ideal for the generation of positively charged C-terminal peptides that are compatible with LC-MS/MS. LysN also functions as a sister enzyme to LysC, which preferentially hydrolyzes C-terminal lysine residues (Raijmakers et al. 2010; Zhao et al. 2020).

The aim of this work was to produce a novel LysN peptidase with robust biochemical characteristics that is applicable over a broad pH range. The recombinant LysN presented in this study was identified in *Trametes coccinea* BRFM310*.* The LysN from *T. coccinea* BRFM310 (named “*Tc*-LysN”) was recombinantly expressed in *Komagataella phaffii* and purified to homogeneity by a single-step anion-exchange chromatography*. Tc*-LysN was biochemically characterized and the mechanism involved in its maturation was also evaluated.

## Materials and methods

### Chemicals and equipment

Analytical grade reagents and chemicals were procured from either Merck (Darmstadt, Germany) or Carl Roth (Karlsruhe, Germany) unless stated otherwise. Azocasein was obtained from Megazyme (Limerick, Ireland). Spectrophotometric analyses were carried out in Epoch 2, manufactured by Biotek (Winooski, USA). Flat-bottom 96-well microtiter plates were purchased from Carl Roth (Karlsruhe, Germany). SDS-PAGE was carried out in Mini Gel Tank by Thermo Fisher Scientific (Dreieich, Germany). All the equipment for nano-LC-ESI-MS/MS experiments was manufactured by Thermo Fisher Scientific (Dreieich, Germany). Protein purification via liquid chromatography was carried out on Äkta Go manufactured by GE Healthcare Biosciences (Uppsala, Sweden). Reaction vessels were incubated in ThermoMixer® C manufactured by Eppendorf (Hamburg, Germany).

### Gene fragment, plasmid, strains, media, and kits

The native pro-protein (zymogen) sequence of *Trametes coccinea*’s peptidyl-lys metalloendopeptidase (UniProt accession# A0A1Y2IQZ5) was back-translated and codon optimized for expression in *Komagataella phaffii*. The gene fragment was ordered for synthesis at Twist Bioscience (San Francisco, CA, USA). The proprietary expression vector, pBSY2S1Z (plasmid map can be found in Online Resource 1), and the proprietary expression host, *Komagataella phaffii* BG10, were procured from BISY (Graz, Austria). *E. coli* DH5α was purchased from New England Biolabs (Frankfurt am Main, Germany). Invitrogen’s *Pichia* EasyComp™ Transformation Kit (Thermo Fisher Scientific, Dreieich, Germany) was used to prepare and transform competent *Komagataella phaffii* BG10 cells. Restriction digestion enzymes, ligase(s), and buffer(s) were sourced from New England Biolabs (Frankfurt am Main, Germany). All media, including Luria-Bertani (LB), yeast extract peptone dextrose (YPD), buffered glycerol complex (BMGY), and buffered methanol complex (BMMY), were prepared according to the guidelines of Invitrogen’s *Pichia* Expression Kit (Publication # MAN0000012). Zeocin® was purchased from Invivogen (Toulouse, France). Molecular biology kits, including plasmid miniprep, DNA purification, and gel extraction, were sourced from Zymo Research Europe (Freiburg im Breisgau, Germany).

### Construction of pBSY2S1Z—*Tc*-LysN plasmid and expression of *Tc*-LysN

The native pro-protein gene for *Tc*-LysN was cloned in-frame with the *S. cerevisiae*’s α-mating factor secretory signal into pBSY2S1Z, under the control of the methanol-inducible AOX1 promoter, via golden gate cloning (Engler et al. 2008) using SapI restriction sites. Chemically competent *E. coli* DH5α cells were transformed with the resulting expression vector, pBSY2S1Z–*Tc*-LysN. Isolated recombinant plasmids from single-colony transformants, selected on low salt Luria-Bertani (LB) plates supplemented with 25 μg*mL^−1^ Zeocin®, were sent for DNA sequencing to Genewiz (Leipzig, Germany).

*Komagataella phaffii* BG10 transformation was carried out by stringently following the guidelines of Invitrogen’s *Pichia* EasyComp™ Transformation Kit. In brief, 50 μL chemically competent *Komagataella phaffii* BG10 cells were transformed using ∼10 μg purified pBSY2S1Z–*Tc*-LysN that was linearized by SacI-HF (according to NEB’s protocol). Single colonies were screened for enzyme activity after ~24 h of induction in BMMY at 30 °C using the standard method outlined in Invitrogen’s *Pichia* EasyComp™ Transformation Kit. *Tc*-LysN activity was analyzed by employing the azocasein assay (“Determination of endopeptidase acting using the azocasein assay” section).

### Quantification of protein content

RotiNanoquant 5X (Carl Roth, Karlsruhe, Germany) was used to estimate protein content based on the method established by (Bradford 1976). In short, 50 μL sample and/or standard was mixed with 200 μL RotiNanoquant (1X) and incubated at 30 °C for 5 min in dark. The absorbance at 450 nm and 590 nm was measured using the microtiter plate reader. A calibration curve was generated using bovine serum albumin (BSA; Biowest, Nuaillé, France) as the standard protein within the range of 0–150 μg*mL^−1^.

### Sodium dodecyl sulfate polyacrylamide gel electrophoresis (SDS-PAGE) and molecular mass estimation

Laemmli’s (1970) protocol was employed for SDS-PAGE with minor modifications. A gradient gel (4–20%; Novex™ WedgeWell™ Tris-Glycine, Thermo Fisher Scientific GmbH, Dreieich, Germany) was used to separate proteins. Broad range protein markers (10–200 kDa; P7719S, New England Biolabs GmbH, Frankfurt am Main, Germany) were utilized as reference proteins for molecular mass estimation. Protein bands were visualized by Coomassie staining using GelCode™ Blue Safe dye (Thermo Fisher Scientific GmbH, Dreieich, Germany).

### In-gel digestion

In-gel digestion of SDS-PAGE bands was performed using trypsin (Roche, Germany) according to the method of Shevchenko et al. (1996). Following enzymatic digestion, the resulting supernatant was transferred to a fresh tube, subjected to drying using a vacuum centrifuge, and then preserved at –20 °C. Subsequently, the dried samples were reconstituted in a solution containing 0.1% (v/v) trifluoroacetic acid (TFA) for analysis using nano-liquid chromatography–tandem mass spectrometry (nano-LC-MS/MS). Identification of neo-N-Termini was performed by in-gel labeling of protein N-termini using TMTpro Zero (Thermo Fisher Scientific) according to the manufacturer’s instructions. Briefly, gel bands were excised with a scalpel from the SDS-PAGE gel and cut into small cubes. Gel cubes were completely covered with acetonitrile and incubated for 10 min. Subsequently, the supernatant was removed and gel cubes were covered with 50 mM HEPES pH 8.5 and incubated for 10 min. This procedure was repeated three times to completely remove the SDS/Tris-HCl buffer from the gel cubes. TMTpro Zero labeling was performed by adding 10 μL TMTpro Zero reagent (10 μg*μL^−1^ in HEPES pH 8.5) and 150 μL of 50 mM HEPES pH 8.5 to the sample, followed by incubation for 1 h at room temperature. The labeling reaction was stopped by adding 15 μL hydroxylamine (5% v/v). Reduction, alkylation, and further processing of the gel cubes was performed according to the reference protocol of Shevchenko et al. (1996), with the exception that trypsin and chymotrypsin (Roche, Penzberg, Germany) were used as peptidases.

### In-solution digestion of bovine serum albumin (BSA) using *Tc*-LysN

Two microgram bovine serum albumin (Sigma-Aldrich, Taufkirchen, Germany) was dissolved in 2 M urea, 10 mM ammonium hydrogencarbonate (pH 8.8). Dithiothreitol (DTT) was added to a final concentration of 10 mM for reduction of cysteines. The samples were then incubated for 30 min at 37 °C under shaking at 1000 rpm. Alkylation of cysteines was performed by adding 30 mM iodoacetamide, followed by incubation at 37 °C for 20 min in the dark. Alkylation was stopped by adding 50 mM DTT and the pH was adjusted by adding ammonium acetate (pH 5.0) to a final concentration of 100 mM. At a peptidase:substrate ratio of 1:50, 40 ng *Tc*-LysN peptidase was added and the samples were digested overnight at 40 °C. In a control experiment, BSA was digested overnight with trypsin in 10 mM ammonium hydrogencarbonate (pH 8.8) at 37 °C using a peptidase:substrate ratio of 1:20. Peptide mixtures were concentrated and desalted on C18 stage tips (Rappsilber et al. 2003) and dried under vacuum. Dried samples were dissolved in 30 μL 0.1 % (v/v) TFA and aliquots of 1 μL were injected for nanoLC-MS/MS analyses.

### Nano-LC-MS/MS

Nano-LC-ESI-MS/MS experiments were carried out using an Ultimate 3000 RSLC nano system (Dionex, Thermo Fisher Scientific, Germany) connected to an Orbitrap Exploris 480 mass spectrometer (Thermo Fisher Scientific, Germany) through an EASY-Nano Flex ion source (Thermo Fisher Scientific, Germany). Tryptic peptides were injected directly to a pre-column (μ-pre-column C18 PepMap100, 300 μm, 100 Å, 5 μm × 5 mm, Thermo Fisher Scientific) that was connected to a NanoEase analytical column (NanoEase M/Z HSS C18 T3, 1.8 μm, 100 Å, 75 μm × 250 mm column, Waters GmbH, Germany). The columns were operated at 35 °C. Flow rate during gradient elution was maintained at 300 nL*min^−1^. An LC-gradient with the following profile was implemented: 2–55% solvent B in 27 min, 55–95% solvent B in 10 min, 5 min isocratic at 95% solvent B, 95–2% solvent B in 10 min, re-equilibration for 5 min at 2% solvent B. Solvent A was 0.1% (v/v) formic acid in H_2_O. Solvent B was 0.1% (v/v) formic acid in acetonitrile. XCalibur version 4.4 (Thermo Fisher Scientific Inc., USA) controlled the Orbitrap Exploris 480. Survey spectra (m/z = 300–1500) were detected in the Orbitrap at a resolution of 60.000 at *m*/*z* = 200. Data-dependent MS/MS mass spectra were generated for the 30 most abundant peptide precursors using high energy collision dissociation (HCD) fragmentation at a resolution of 15,000 with normalized collision energy of 30.

### MS data analysis

Proteins were identified in Mascot 2.6 (Matrix Science, UK). Spectra were searched against the Swissprot database or the *Trametes coccinea* BRFM310 protein database downloaded as FASTA-formatted sequences from UniProt (www.uniprot.org). Search parameters specified LysN as cleaving enzyme, a 5-ppm mass tolerance for peptide precursors, and 0.02 Da for fragment ions. Alternatively, no search enzyme was specified for an unspecific search. Carbamidomethylation of cysteine residues was defined as fixed modification. Methionine oxidation was allowed as variable modification. For TMTpro Zero experiments trypsin and chymotrypsin were specified as cleaving enzymes and TMTpro Zero was allowed as variable modification at peptide N-termini and lysine. Mascot search results were imported into Scaffold version 4.10.0. (Proteome Software, USA). Peptide identifications were accepted with a peptide probability greater than 90.0% as specified by the Peptide Prophet algorithm (Keller et al. 2002). Proteins had to be identified by at least two peptides and a protein probability of at least 99% to be accepted. Protein probabilities were assigned by the Protein Prophet algorithm (Nesvizhskii et al. 2003).

### Production of *Tc*-LysN in shake-flasks

*Tc*-LysN was produced inside a 2 L baffled shake-flask containing 200 mL BMMY, by adapting a high-cell density fermentation method (Kaushik et al. 2016). In short, a preculture of recombinant *K. phaffii* was cultivated overnight at 30 °C in 50 mL BMGY (containing 100 μg*mL^−1^ zeocin). The preculture was used to inoculate 200 mL of BMGY inside a 2 L baffled shake-flask at 30 °C and 180 rpm until the OD_600nm_ reached ~47.0. The cells were then pelleted and resuspended in 200 mL BMMY (containing 0.5% methanol) inside a 2 L baffled shake-flask. *Tc-*LysN expression was induced for 96 h at 30 °C and 180 rpm with 0.5% methanol supplementation every ~12 h. At the end of the induction phase, the culture medium was centrifuged at 5000 × g and the supernatant was filtered through a 0.22 μm membrane. The supernatant was then concentrated ~20-fold using a 5 kDa cross-flow membrane (Vivaflow® 50 PES, Sartorius Stedim, Goettingen, Germany) on ice. The concentrated *Tc*-LysN was then buffer exchanged with 20 mM MOPS buffer (pH 7.2) using the same cross-flow membrane.

### Purification of *Tc*-LysN

Protein purification was performed at room temperature on the Äkta Go purification system. The filtered, concentrated, and buffer-exchanged *Tc*-LysN was loaded onto a HiTrap Q FF 5 mL column (GE Healthcare Bio-Sciences, Munich, Germany) via the sample injection pump. The column was pre-equilibrated with the binding buffer (20 mM MOPS pH 7.2). Sample was applied at a flowrate of 2 mL*min^−1^. Unbound protein was washed out with 22 column volumes of binding buffer at a flowrate of 10 mL*min^−1^. The elution was carried out with a linear gradient (0–65%) using the elution buffer (20 mM MOPS + 1 M NaCl pH 7.2) at a flowrate of 2 mL*min^−1^. Protein detection was monitored at 280 nm. Fractions were tested for *Tc-*LysN activity using the azocasein assay. Fractions with the highest *Tc-*LysN activity were pooled and then dialyzed against 5 mM sodium acetate buffer (pH 5.0). Aliquots of the dialyzed *Tc-*LysN were stored at –20 °C until further use.

### Activation of *Tc*-LysN zymogen

In order to investigate the potential involvement of an endogenous *K. phaffii* endopeptidase with trypsin-like activity in the maturation of *Tc*-LysN zymogen into its active form, 250 μL of concentrated BMMY culture supernatant (post 24 h induction) was incubated with 425 USP-U of porcine pancreatic trypsin (Carl Roth, Karlsruhe, Germany) at pH 7.5 (60 mM MOPS) for 60 min at 37 °C. To account for any changes induced simply because of temperature and/or prolonged incubation time, two controls were also set-up: one with only culture supernatant without trypsin and another with only trypsin and no culture supernatant. Samples were withdrawn at specified time points to prepare for SDS-PAGE analysis and assessment of *Tc*-LysN’s activity. Samples for SDS-PAGE analysis were immediately mixed with reducing sample loading buffer and heated for 5 min at 90 °C before being loaded onto a 4–20% precast gradient gel (Novex™ WedgeWell™ Tris-Glycine, Thermo Fisher Scientific GmbH, Dreieich, Germany). Samples withdrawn for *Tc*-LysN’s activity measurement were first incubated with 10 mM phenylmethylsulfonyl fluoride (PMSF; Carl Roth, Karlsruhe, Germany) for 30 min at room temperature to inactivate trypsin. The experiment was additionally conducted with varied parameters such as trypsin dose, incubation time, and incubation temperature.

### Biochemical characterization

#### Determination of endopeptidase acting using the azocasein assay

The azocasein assay was performed to determine the proteolytic activity of *Tc*-LysN according to the method of Iversen and Jørgensen (1995) with slight modifications (Ahmed et al. 2022). Substrate stock solution, 3% (w/v), was prepared by dissolving azocasein in H_2_O_dd_. The assay was performed as follows: 200 μL of sodium acetate buffer (pH 5.0, 50 mM final concentration) and 30 μL of the azocasein stock solution was added to a 1.5 mL microfuge tube. The substrate was equilibrated within the specified temperature range (37 °C–90 °C) for 5 min. The hydrolysis was initiated by adding 10 μL of appropriately diluted and separately pre-equilibrated (37 °C–90 °C) purified *Tc*-LysN. The hydrolysis was carried out within the specified temperature range (37 °C–90 °C) in a thermo mixer at 1000 rpm. The hydrolysis was terminated at various time intervals by dispensing 30 μL of 2 M tricholoroacetic acid (TCA). For blanks, 30 μL of 2 M TCA was added prior to the addition of enzyme under the same conditions. The spectrophotometric analysis was carried out by dispensing 150 μL of 1 M NaOH into microtiter plate wells, followed by 150 μL supernatant from the centrifuged hydrolysates. The absorbance was measured at 450 nm using the microtiter plate reader after 15 s of linear shaking at room temperature. One azocasein unit (ACU) was defined as the increase of 1 absorbance unit*min^−1^*mL^−1^ at 450 nm.

#### Determination of pH optimum, temperature maximum, and thermostability

The azocasein assay was employed to characterize *Tc*-LysN biochemically. The pH optimum was determined by measuring the proteolytic activity after 5 min at 37 °C. Buffers (50 mM final concentration) with overlapping pH range (sodium citrate-citric acid pH 3.0–4.0; sodium acetate pH 4.0–5.5; MES pH 5.5–6.6; MOPS pH 6.6–7.5; Tris-HCl pH 7.5–8.5; glycine-HCl pH 8.5–10) were utilized to simultaneously evaluate the effect of buffer salts on proteolytic activity. The temperature-maximum was determined by measuring proteolytic activity at pH 5.0 in 50 mM (final concentration) sodium acetate buffer within the temperature range of 10–90 °C after 10 min. Additionally, aliquots of the purified *Tc*-LysN were incubated for ~18 h at temperature intervals between 0 °C—80 °C. The thermostability of *Tc*-LysN was evaluated by measuring the proteolytic activity at 60 °C in sodium acetate buffer (final concentration 50 mM, pH 5.0) using the aforementioned pre-incubated *Tc*-LysN aliquots. Proteolytic activities were calculated as averages of triplicate measurements for each experiment.

#### Effect of ions, solvents, reducing agents, and peptidase inhibitors on proteolytic activity

The azocasein assay was employed to determine the effect of mono/divalent ions, solvents, reducing agents, and peptidase inhibitors at various concentrations on the proteolytic activity of the purified *Tc*-LysN. The assay was carried out in sodium acetate buffer (50 mM final concentration, pH 5.0) at 60 °C. The purified *Tc*-LysN was incubated with the respective test substances at final concentrations of 5 mM and/or 10 mM for 10 min at 60 °C before initiating the hydrolysis by the addition of the pre-equilibrated (60 °C) substrate. Proteolytic activity observed without the addition of any test substance was defined as 100% activity. Proteolytic activities were calculated as averages of triplicate measurements for each experiment.

#### GenBank accession numbers

The GenBank accession number for the primary amino acid sequence of peptidyl-lys metalloendopeptidase from *T. coccinea* BRFM310 (*Tc*-LysN) is OSD03546.1. The GenBank accession number for the codon-optimized synthetic nucleotide sequence used to express *Tc*-LysN in *K. phaffii* is OR161067.

## Results

### Production of *Tc*-LysN in *K. phaffii*, its purification and activation

**Fig. 1:**
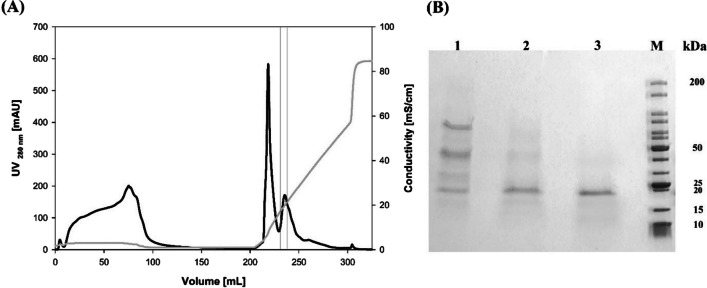
Anion exchange (HiTrap QFF 5 mL) chromatogram (**A**) of concentrated BMMY supernatant after ~96 h of induction with methanol. Pooled fractions with *Tc*-LysN activity are enclosed within two straight lines. Sodium dodecyl sulfate–polyacrylamide gel electrophoresis (**B**) of the *Tc*-LysN purified from culture supernatant via AEX. Lane M contains the marker proteins (broad range 10–200 kDa, NEB). Lane 1 contains culture supernatant. Lane 2 contains the AEX fractions that were collected at the trailing end of peak enclosed within two straight lines in 1A. Lane 3 contains the pooled active fractions from AEX (enclosed within two straight lines in the chromatogram). Protein load was ~2 μg protein per lane. SDS-PAGE image with enhanced contrast can be found in Online Resource 2

*Tc*-LysN was expressed as pre-pro-protein using the native pro-protein sequence and *S. cerevisiae*’s α-mating factor secretory signal. *Tc*-LysN was secreted into BMMY culture broth as pro-*Tc*-LysN (zymogen) as well as *Tc*-LysN (active enzyme) upon induction with 0.5% methanol every 12 h for up to 96 h. No significant change in the band intensities of pro-*Tc*-LysN and *Tc*-LysN was observed on SDS-PAGE after ~44 h of induction (data not shown) which suggested that pro-*Tc*-LysN matured into its active form intracellularly. The filter-sterilized culture broth was concentrated using a 5 kDa cross-flow membrane. The concentrated culture broth was buffer-exchanged with 20 mM MOPS buffer (pH 7.2) and applied onto HiTrap Q FF 5 mL column for anion-exchange chromatography (AEX). Fractions with proteolytic activity eluted between 16 and 22 mS*cm^−1^ (Fig. 1A). The purified mature *Tc*-LysN migrated as a single band of ~19.8 kDa on SDS-PAGE and pro-*Tc*-LysN from the culture supernatant migrated as a band of ~38 kDa (Fig. 1B and Online Resource 2). The concentration of the purified *Tc*-LysN was ~1.3 mg*L^−1^ as determined by the Bradford assay. Total enzyme activity of ~40 ACU was obtained from the culture broth at the end of purification with an activity yield of ~9%.

**Fig. 2:**
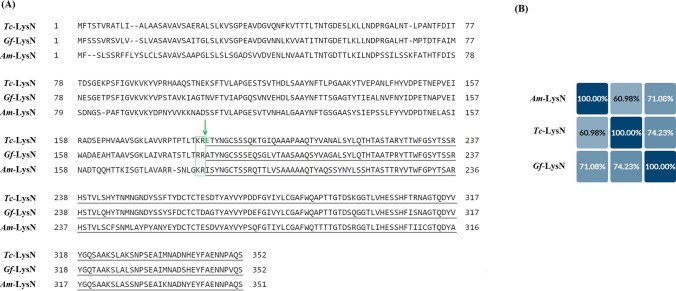
Multiple sequence alignment (**A**) of LysN peptidases from *Trametes coccinea*, *Grifola frondosa*, and *Armillaria mellea*. Amino acid sequences of the mature LysN peptidases are underlined in gray. The Kex2 recognition sites (KR and RR) between the pro-peptides and mature proteins are boxed in green and the cleavage site is indicated by a green arrow. The TMTpro Zero labeled N-terminus amino acid, E185, of *Tc*-LysN is typed in green. Percent identity matrix (**B**) shows >50% homology across the three mature LysN peptidases

For identity verification, the ~19.8 kDa band of purified protein (Fig. 1B and Online Resource 2) was subjected to in-gel digestion with trypsin followed by nano-LC–ESI–MS/MS analysis. The ~19.8 kDa band was identified as peptidyl-lys metalloendopeptidase from *Trametes coccinea* (Uniprot Accession Number A0A1Y2IQZ5). Nano-LC-ESI-MS/MS analysis revealed a 40% sequence coverage of the *Tc*-LysN precursor protein sequence by tryptic peptides. No tryptic peptides from the N-terminus of the precursor protein, including the potential pro-peptide, were identified. The predicted amino acid sequence of the *Tc*-LysN zymogen was aligned with the annotated amino acid sequences of *Gf-*LysN zymogen and *Am*-LysN zymogen using National Center for Biotechnology Information (NCBI)’s constraint-based multiple alignment tool (COBALT). The mature *Tc*-LysN shares 60.98% homology with mature *Am-*LysN and 74.23% homology with mature *Gf*-LysN (Fig. 2B). To identify the N-terminus of the active *Tc*-LysN peptidase, the ~19.8 kD band of mature *Tc*-LysN was labeled in-gel using TMTpro Zero reagent and the gel band was subsequently digested with trypsin and chymotrypsin. TMTpro Zero reacts with alpha amino groups of intact proteins and lysine amino acid side chains. Therefore, peptides with a labeled alpha amino group should comprise the protein N-terminus (Kleifeld et al. 2010). Nano-LC-MS/MS analysis of the tryptic and chymotryptic peptides (Online Resource 3) revealed that peptides labeled with TMTpro Zero at the alpha amino group contained Glu185 as the N-terminal amino acid, which indicated that the active *Tc*-LysN peptidase might be produced by removing the first 184 amino acids (Fig. 2A). This finding suggested that the cleavage of the pro-peptide occurred at the Kex2 cleavage site (KR↓), which is natively present in the pro-*Tc*-LysN (Fig. 2A). To assess whether or not a trypsin-like endogenous *K. phaffii* endopeptidase was involved in the cleavage of *Tc*-LysN pro-peptide, the culture supernatant was incubated with porcine pancreatic trypsin under varied experimental conditions and samples were collected at specified time intervals to evaluate the effect on *Tc*-LysN’s activity and behavior on SDS-PAGE. The bands of *Tc*-LysN zymogen and mature *Tc*-LysN on SDS-PAGE remained unchanged over the course of 60 mins incubation with catalytic amount of trypsin (Online Resource 4). Also, no increase in *Tc*-LysN’s activity in the culture supernatant was observed. Similar results were obtained when this experiment was repeated with variations is parameters such as trypsin dose, incubation time, and incubation temperature (data not shown). It was, thus, concluded that an endogenous *K. phaffii* peptidase with trypsin-like activity is not involved in the activation of the *Tc*-LysN zymogen. The molecular weight, based on the identified N-terminus of the active *Tc*-LysN, was calculated be 18.3 kDa, which is in close agreement with the molecular weight deduced from the band on SDS-PAGE (Fig. 1B).

### Biochemical characterization

**Fig. 3:**
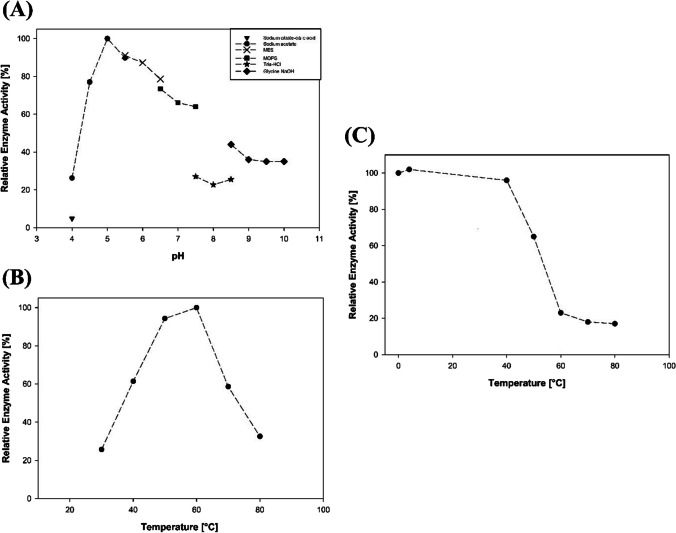
pH-optimum (**A**) of *Tc*-LysN using different buffers (50 mM) with overlapping pH range. Temperature-maximum (**B**) of *Tc*-LysN determined at pH 5.0 in 50 mM sodium acetate buffer. Thermostability (**C**) of *Tc*-LysN after 18 h of incubation at specified temperatures. All data points are averages of triplicate measurements; the standard deviation was <5%

The proteolytic activity of *Tc*-LysN under various pH and temperature conditions as well as in the presence of chaotropes, organic solvents, and different divalent cations was evaluated using azocasein as substrate. Buffers with overlapping pH range were used to simultaneously analyze the effect of buffer salts on proteolytic activity. *Tc*-LysN exhibited maximum activity at 60 °C in 50 mM sodium acetate at pH 5.0 (Fig. 3A and 3B), while it maintained >50% activity between 40 °C—70 °C. *Tc*-LysN maintained >60% activity between pH 4.5—7.5 and 30%—40% activity between pH 8.5—10.0. *Tc-*LysN’s thermostability was evaluated by testing the proteolytic activity of *Tc*-LysN aliquots that had been incubated for ~18 h at various temperatures under optimum assay conditions. Figure 3C summarizes the effect of various temperatures on the thermostability of *Tc*-LysN after 18 h of incubation. *Tc*-LysN maintained ~65% activity after 18 h of incubation at 50 °C and even retained ~20% activity after being incubated at 80 °C for 18 h.

The influence of different divalent cations, solvents, and denaturing agents on *Tc*-LysN’s proteolytic activity was studied to characterize its performance under variable sample preparation conditions. The results are summarized in Table 1. *Tc*-LysN’s activity was found to be enhanced by high concentrations of organic solvents. Acetonitrile at a concentration of 40% (v/v) enhanced *Tc*-LysN’s activity up to ~100%, whereas 40% (v/v) methanol increased the activity up to ~50%. Up to 8 M urea had no significant negative effect on *Tc*-LysN’s activity. In contrast, >1 M guanidine hydrochloride was not tolerated by *Tc*-LysN and the activity was reduced down to ~30% in its presence. With the exception of 5 mM Cu^2+^, which reduced *Tc*-LysN’s activity down to ~20%, none of the other divalent cations (up to 10 mM) affected *Tc*-LysN’s activity negatively. Cobalt at 5 mM was found to enhance *Tc*-LysN’s activity up to ~60%. Among peptidase inhibitors, only 33.33 mM EDTA was found to completely inactivate *Tc-*LysN after overnight incubation at 4 °C.

**Table 1:** Effect of organic solvents, cations, denaturing agents, metal chelators, and peptidase inhibitors on the activity of the purified *Tc*-LysN (at 60 °C in 50 mM sodium acetate, pH 5.0). The enzyme was assayed using azocasein as substrate

	Substance	Concentration	Activity [%]^1^	Substance	Concentration	Activity [%]^1^
Solvents [% (v/v)]	Acetonitrile	20	214 ± 1.94	Methanol	20	164 ± 1.77
		40	202 ± 11		40	151 ± 8.1
Cations [mM]	Co^2+^	5	161 ± 3.6	Cu^2+^	5	20.52 ± 12.6
		10	116.12 ± 1.99		10	c.n.d.
	Zn^2+^	5	105 ± 3.44			
		10	89 ± 8.1			
	Mg^2+^	5	122 ± 0.73			
		10	116.58 ± 0.12			
Reagents [mM]	*β*-mercaptoethanol	10	114 ± 9.8	Urea	1000	96 ± 11
					2000	94 ± 1
					4000	90 ± 9.5
	DTT	10	90 ± 11		6000	89 ± 5.6
					8000	94 ± 5.8
	EDTA	10	30 ± 4.4	Guanidine HCl	1000	77 ± 4
		33.33^*^	0 ± 0.78		2000	23 ± 11
					4000	15 ± 2.3
					6000	39 ± 8.4
					8000	43 ± 2.7
Peptidase inhibitors [mM]	E-64	1	105.25 ± 5.9	Pepstatin a^2^	0.1	120 ± 5.6
	Aprotinin	0.1	99.51 ± 4			

^1^The activity determined in the absence of test substances was defined as 100% activity. ^2^Dissolved in DMSO. All the substances were dissolved in H_2_O_dd_ unless stated otherwise. ^*^Tc-LysN had to be incubated overnight at 4 °C to observe this inhibition. The values reported are the averages of triplicate measurements

### In-solution digestion of bovine serum albumin (BSA) with Tc-LysN

*Tc*-LysN was evaluated for its application in proteomics experiments. Bovine serum albumin (BSA), a typical standard protein used in proteomics workflows, was used as substrate. Two microgram BSA was digested in solution with *Tc*-LysN at a peptidase:substrate ratio of 1:50. The digestion was carried out overnight at 40 °C using a volatile ammonium acetate buffer pH 5.0. The subsequent nano-LC-MS/MS analysis revealed 84% sequence coverage of BSA with *Tc*-LysN peptides (Online Resource 5). In a control experiment using trypsin as peptidase, 90% sequence coverage of BSA was observed (data not shown). However, the number of identified peptides (115 unique peptides) was higher compared to the *Tc*-LysN digest (78 unique peptides). These experiments indicate that *Tc*-LysN may be suited for proteomics experiments equally well as established peptidases like trypsin.

## Discussion

Purification of LysN peptidases from the fruiting bodies of organisms such as *Grifola frondosa* is a labor- and resource-intensive procedure as demonstrated by Nonaka et al. (1995), Stressler et al. (2014), and more recently by Zhao et al. (2020). Recombinant expression of LysN simplifies the downstream processing significantly.

### Production of *Tc*-LysN in *K. phaffii*, its purification and activation

The nano-LC-ESI-MS/MS analysis identified *Tc*-LysN as peptidyl-lys metalloendopeptidase from *Trametes coccinea* (Uniprot accession number A0A1Y2IQZ5). The N-terminal sequencing of the mature *Tc*-LysN indicated that the likely route of activation of *Tc*-LysN zymogen is the processing of the Kex2 site (KR) by an endogenous *K. phaffii* Kex2 peptidase or by another, as yet unknown, endogenous *K. phaffii* endopeptidase with trypsin-like activity. The latter hypothesis was tested by using trypsin to induce maturation of the *Tc*-LysN zymogen. In any applied dosage, trypsin was found to be unable to activate the *Tc*-LysN zymogen within the given incubation period (1–6 h). This result appears to be in line with Ødum et al*.*’s (2016) findings about recombinant *Am*-LysN also expressed in *K. phaffii*—with the notable exception that they were able to isolate only the mature LysN from their culture medium, while in this study, the presence of both, the LysN zymogen and the mature LysN, was detected. This could likely be explained by the fact that in this study, *S. cerevisiae*’s α-mating factor was linked to the native pro-protein gene for *Tc*-LysN by a Kex2 cleavage site. The competition for cleavage at the two Kex2 sites (between α-mating factor and pro-protein and between pro-peptide and mature protein) could have resulted in limited maturation of the *Tc*-LysN zymogen.

The observed MW of *Tc*-LysN is consistent with the MWs of previously studied LysN peptidases (Wingard et al. 1972; Nonaka et al. 1995; Saito et al. 2002; Stressler et al. 2014; Ødum et al. 2016a). The *Tc*-LysN activity obtained at the end of purification was ~40 ACU under optimal conditions, which corresponded to an activity yield of ~9%. Due to the unavailability of data about enzyme activities of recombinant LysN peptidases reported in other studies, a comparison between enzyme activities could not be made. The concentration of the purified *Tc*-LysN was ~1.3 mg*L^−1^, which is 5.2 times higher than the hexa-histidine-tagged recombinant *Am*-LysN expressed in *K. phaffii* (Ødum et al. 2016)*.* The use of a 5 kDa membrane for cross-flow filtration during downstream processing could have resulted in some loss of the ~19.8 kDa *Tc-*LysN resulting in the reduced final yield. The recombinant *Am-*LysN was purified to the final concentration of ~0.25 mg*L^−1^ from minimal glucose medium, but data about its activity yield were not reported (Ødum et al. 2016)*.* The hexa-histidine-tagged *Gf*-LysN from *G. frondosa* was also recombinantly expressed in *K. phaffii*, but data about its activity yield and final concentration were not reported (Saito et al. 2002). *My-*LysN was purified from the culture medium of *Myxobacter* AL-1 with the final activity yield of ~4% after 12 purification steps (Wingard et al. 1972). *Gf*-LysN was purified from the fruiting bodies of *G. frondosa* in four steps with the final activity yield of ~25% (Nonaka et al. 1995). *Gf-*LysN was also partially purified from the fruiting bodies of *G. frondosa* to the final concentration of 2 mg*L^−1^ in three purification steps with the final activity yield of ~0.2% (Stressler et al. 2014). More recently, *Gf-*LysN was purified from the fruiting bodies of *G. frondosa* to the final concentration of 500 mg*L^−1^ in six purification steps with the final activity yield of ~0.6% (Zhao et al. 2020). A valid comparison between the yields of recombinant LysN peptidases and yields of LysN peptidases purified from homogenates of the fruiting bodies of the basidiomycetes could not be made since the dry mass of the fruiting bodies and its initial processing varied significantly from one study to another.

### Biochemical characterization

Using the model substrates, azocoll or azocasein, *My*-LysN, *Gf*-LysN, and *Am*-LysN were reported to possess a neutral to alkaline pH optimum. *Gf*-LysN was reported to have a pH optimum of 9.5 with azocasein as substrate (Nonaka et al. 1995), while Stressler et al. (2014) reported <15% azocaseinolytic activity below pH 7.0 for *Gf*-LysN. The LysN isolated from *Pleurotus ostreatus* (*Po*-LysN) was reported to have a pH optimum of 5.6 with azocasein as substrate; however, this was revised to pH 8.5 later (Dohmae et al. 1995). Data about *Po*-LysN’s operational pH range was not made available. *Tc*-LysN exhibited the highest activity in 50 mM sodium acetate pH 5.0 (100%; Fig. 2A) with azocasein as substrate while it retained >60% azocaseinolytic activity between pH values 4.5—7.5. Below pH value 4.5 and above pH value 9.0, the proteolytic activity decreased moderately (>20%<40% proteolytic activity), indicating that *Tc*-LysN is active within a broader pH range when compared to *Gf*-LysN. It should be noted that *Tc*-LysN maintained >30% activity between pH values 9.0—10.0, indicating that it is still applicable at alkaline pH using prolonged incubation.

To the best of our knowledge, *Tc*-LysN is the first peptidyl-lys metalloendopeptidase being reported to work optimally within the acidic pH range while still maintaining proteolytic activity up to pH 8.5 (>40% enzyme activity). *Gf-*LysN and trypsin work optimally within the alkaline pH range; the latter is reversibly inactivated below pH 4.0. The broader operational pH range of *Tc-*LysN could enable the exploration of acidic buffer systems for MS-based proteomics applications. For example, *Tc*-LysN could be ideally suited for disulfide mapping experiments due to its acidic pH optimum at pH 5.0. Sample digestion at pH 5.0 prevents disulfide exchange reactions that usually take place at alkaline pH. Avoiding disulfide exchange reactions might lead to a lower number of false positive identified disulfide bridges (Tsai et al. 2013).

Except for *Gf*-LysN reported by Stressler et al. (2014), which had a temperature maximum of 55 °C, explicit data about temperature maxima for the other LysN peptidases could not be found. All LysN peptidases, however, were reported to be pH and temperature stable (Lewis et al. 1978; Nonaka et al. 1995; Nonaka et al. 1998; Ødum et al. 2016; Wingard et al. 1972). *Tc*-LysN demonstrated maximum proteolytic activity at 60 °C, while maintaining a wide operational range between 40 °C—70 °C (>50% proteolytic activity). *Tc-*LysN also retained 65% proteolytic activity after 18 h of incubation at 50 °C and even retained ~20% activity after 18 h of incubation at 80 °C. These results are comparable to the results reported by Stressler et al. (2014) for *Gf-*LysN*.* As demonstrated by Taouatas et al. (2010), varying the combination of incubation time and reaction temperature can result in different numbers of identified peptides during MS analyses. The wide working pH and temperature range of *Tc*-LysN could, therefore, enable the alteration of experimental parameters within the proteomics workflow with more freedom as compared to trypsin. Currently, trypsin has to be glycated to increase its thermostability to withstand the elevated temperatures during denaturation of native proteins that do not digest readily (Pham et al. 2008). The innate ability of *Tc-*LysN to withstand higher temperatures could make it a more robust proteolytic enzyme to analyze samples that require harsher denaturation steps within the proteomics workflow.

Organic solvents such as acetonitrile and methanol were found to enhance *Tc-*LysN’s activity, where acetonitrile almost doubled the measured azocaseinolytic activity while methanol enhanced the activity up to ~50%. Consistent with the results of Taouatas et al. (2010), high concentrations of guanidine HCl were found to be detrimental to *Tc-*LysN while even 8 M urea had no significant impact on *Tc-*LysN’s proteolytic activity. The exceptional tolerance to high concentrations of urea is notable since higher concentrations of urea can aid the replacement of sodium dodecyl sulfate (SDS), which is used as the primary denaturant in the filter-aided sample preparation (FASP) before MS analysis. High concentrations of urea keep the proteins denatured and soluble when SDS is washed off. Owing to its chaotropic properties, urea also reduces the size of SDS micelles which would otherwise block the pores of the membrane filter (Wis et al. 2009). Reducing agents such as *β*-mercaptoethanol and dithiothreitol (DTT) had no significant inhibitory effect on *Tc*-LysN’s activity even at a concentration of 10 mM, while 1 mM DTT reduced *Gf-*LysN’s activity by 78% (Nonaka et al. 1995). This indicates that potential disulfide groups do not appear to be critical for the proteolytic activity of *Tc*-LysN (Degraeve and Martial-Gros 2003). According to Taouatas et al. (2010), the proteolytic activity of *Gf-*LysN decreased significantly below pH 6.5 and the enzyme was essentially inactivated at pH 3.5. In comparison*,* the *Tc-*LysN reported in this study retained 75%–80% activity at pH 6.5.

Unlike previous reports about LysN peptidases (Nonaka et al. 1995; Stressler et al. 2014; Wingard et al. 1972), *Tc-*LysN was found to be considerably resistant to inactivation by the potent metal chelator, EDTA. Complete inhibition of *Tc-*LysN was only observed after the metalloendopeptidase was incubated with 33.33 mM EDTA overnight at 4 °C, whereas *Gf*-LysN was completely inactivated by 10 μM EDTA (Stressler et al. 2014). In contrast, *Tc*-LysN retained ~30% residual activity after being incubated with 10 mM EDTA at 60 °C for 10 min (data not shown).

Zinc has been reported to be the natural cofactor of *Gf*-LysN and *Am*-LysN. The addition of zinc and other metal ions to apo-*Gf*-LysN has been shown to restore enzymatic activity and also induce changes in thermostability. Contrary to Nonaka et al.’s (1995) findings about *Gf-*LysN, where the addition Co^2+^ reduced *Gf-*LysN’s activity by 40%, *Tc-*LysN’s activity was enhanced by Co^2+^ more than any other divalent metal ion (~161%). It would be of interest to evaluate whether or not the activation of apo-*Tc-*LysN by Co^2+^ and other metal ions could alter *Tc-*LysN’s biochemical properties significantly.

The observed inhibitory effect exerted by Tris-HCl on *Tc-*LysN’s activity could be attributed to the metal ion chelation properties of tris(hydroxymethyl)aminomethane (Tris) due to the presence of primary amines in its structure (Desmarais et al. 2002; Fischer et al. 1979). A similar inhibitory effect of Tris on *Gf*-LysN’s activity at pH 9.0 was reported by Stressler et al. (2014).

Sample preparation remains one of the most crucial initial steps within proteomics workflows. A typical proteomics analysis begins with the proteolytic digestion of all proteins present in a given sample. The resulting peptide mixture is usually separated by chromatography methods and subsequently analyzed by mass spectrometry (LC-MS) (Tsiatsiani and Heck 2015). Even though trypsin is still the most commonly employed peptidase for sample preparation in proteomics workflows, it has some limitations. Reports have indicated that the alkaline conditions optimized for digestion by trypsin cause disulfide bond rearrangement in disulfide mapping experiments (Sanger 1953). This problem can be mitigated by performing the digestion with trypsin at suboptimal pH conditions or, alternatively, by using a peptidase that works well under acidic conditions. It is, therefore, important to explore new peptidases that can handle a wide variety of sample preparation conditions within the proteomics workflow without losing their proteolytic efficiency. *Tc*-LysN was evaluated for its use in proteomics experiments. Bovine serum albumin (BSA) was hydrolyzed with *Tc*-LysN at pH 5.0. The subsequent nano-LC-MS/MS analysis revealed high sequence coverage (84%) of the BSA sequence with *Tc*-LysN peptides (Online Resource 5). Comparable sequence coverage of BSA (90%) was observed when trypsin was used as peptidase in a control experiment (data not shown), however, a higher number of unique peptides was observed. This indicates that *Tc*-LysN may be used in proteomics experiments equally well compared to established peptidases like trypsin. Further experiments will be needed to explore the full potential of *Tc*-LysN for proteomics applications, which are beyond the scope of this manuscript. Both trypsin and *Gf*-LysN are able to tolerate relatively harsh conditions, but both are restricted by their alkaline pH optimum. With its robust biochemical characteristic, its wide working pH range, alongside its acidic pH optimum, *Tc*-LysN could be employed to digest samples where alkaline conditions are not best suited.

## Supplementary information

ESM 1 (PDF 276 kb)

## Data Availability

All data supporting the findings of this study are available within the paper and its accompanying Online Resource. The synthetic gene sequence for *Tc*-LysN was deposited into the GenBank database under accession number *OR161067.*
