# Supplementary material for: A novel, robust peptidyl-lys metalloendopeptidase from Trametes coccinea recombinantly expressed in Komagataella phaffii
Source: Appl Microbiol Biotechnol. 2024 Jan 13;108:103. doi: 10.1007/s00253-023-12986-3 (PMC10787681; doi:10.1007/s00253-023-12986-3)
Supplement: Supplementary file 1 [file 253_2023_12986_MOESM1_ESM.pdf]

A novel, robust peptidyl-lys metalloendopeptidase from *Trametes coccinea* recombinantly expressed in *Komagataella phaffii*

Uzair Ahmed[1,2], Tobias Stadelmann[1], Daniel Heid[1], Berit Würtz[3], Jens Pfannstiel[3], Katrin Ochsenreither[2], and Thomas Eisele[1*]

[1]Hochschule Offenburg, Faculty of Mechanical and Process Engineering, 77652 Offenburg, Germany

[2]Karlsruhe Institute of Technology (KIT), Department of Chemical and Process Engineering, 76131 Karlsruhe, Germany

[3]University of Hohenheim, Mass Spectrometry Unit Core Facility, 70599 Stuttgart, Germany

[*]**Corresponding author:** thomas.eisele@hs-offenburg.de

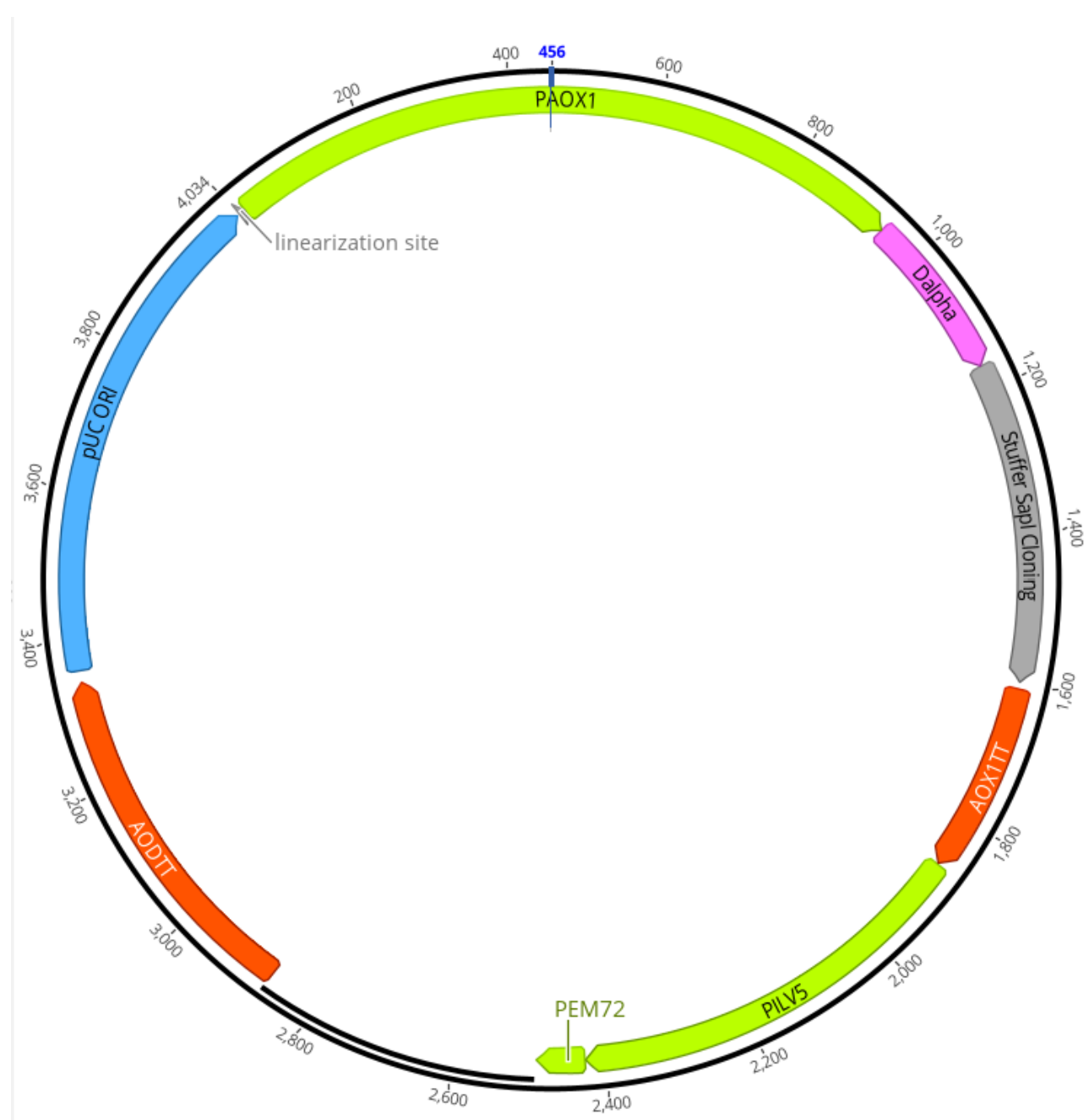

Online resource 1 Plasmid map of pBSY2S1Z.

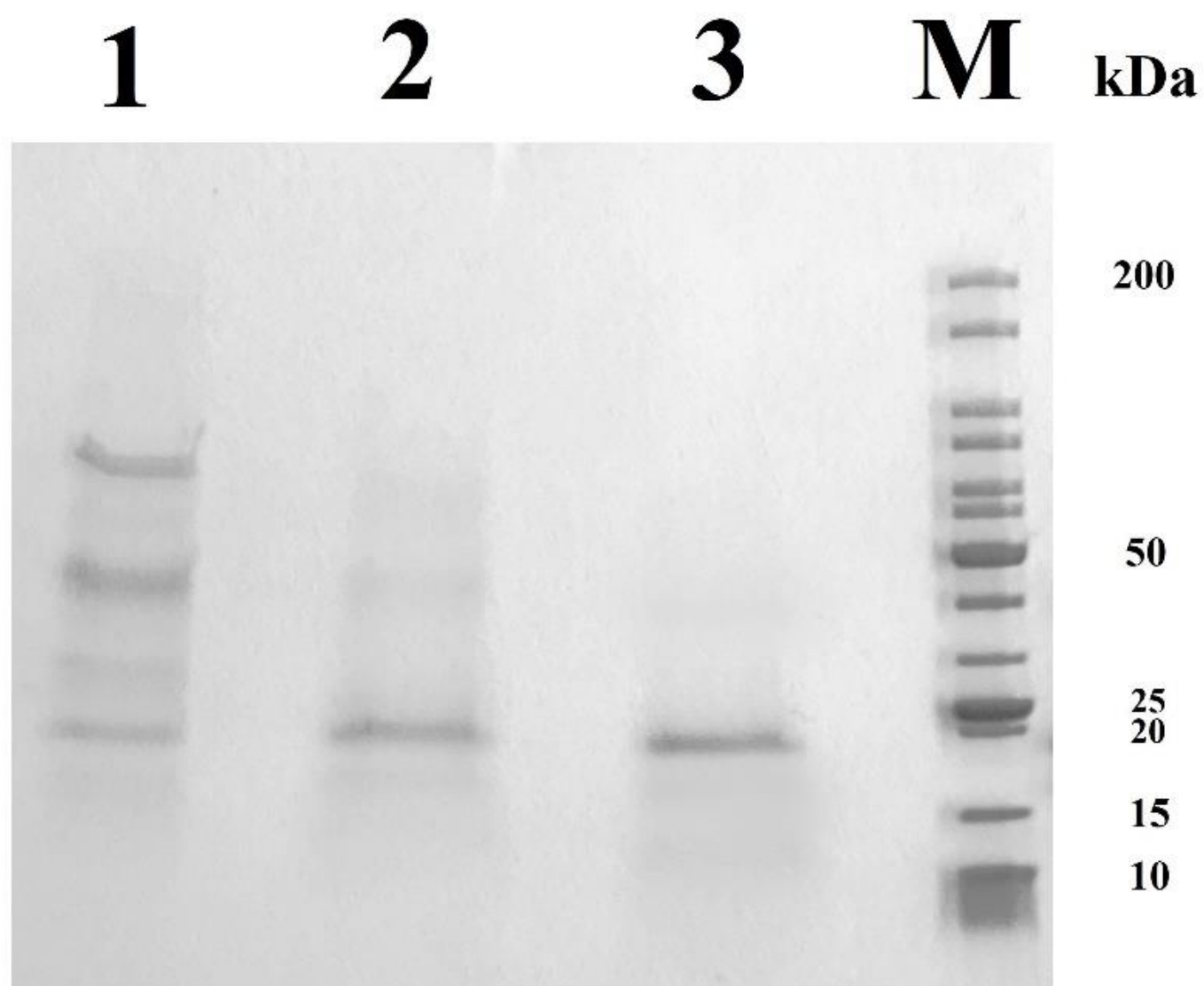

Online resource 2 Sodium dodecyl sulfate–polyacrylamide gel electrophoresis of the *Tc*-LysN purified from culture supernatant via AEX. The image is identical to the Figure 1B in the manuscript; only the contrast has been enhanced.

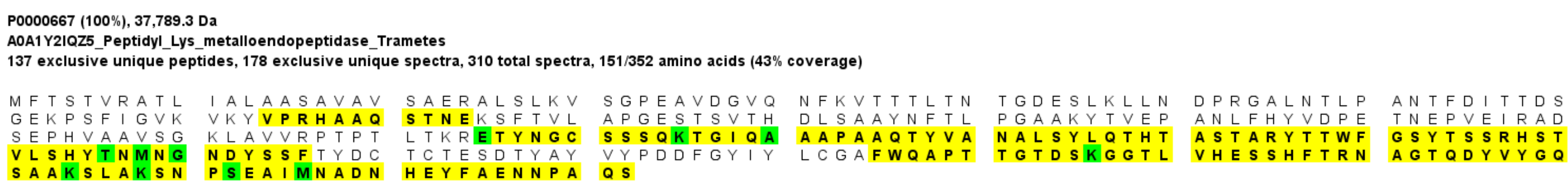

Online resource 3 *Tc*-LysN sequence coverage of TMT-labeling experiment. E185 is the N-terminal TMT-labeled amino acid.

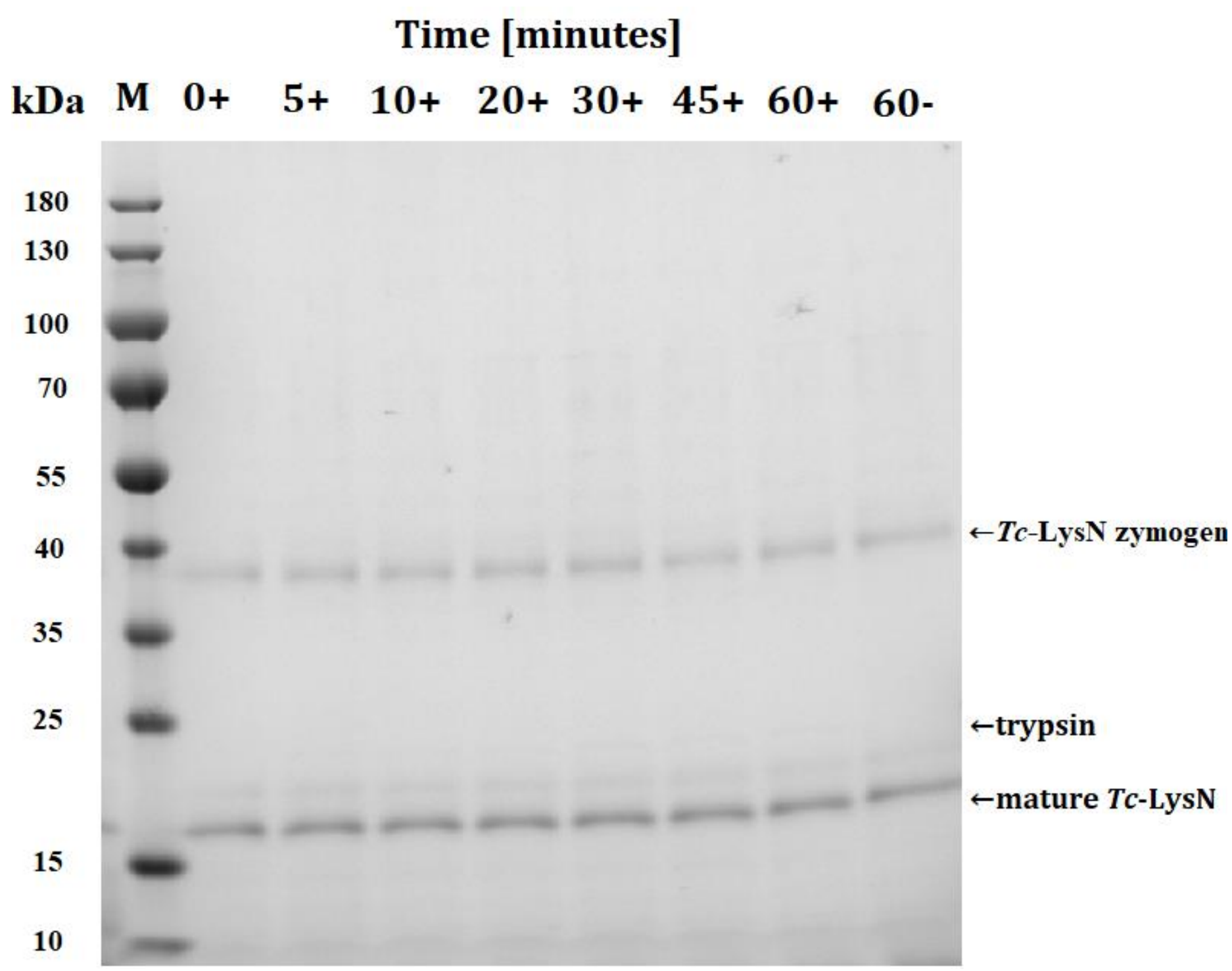

Online resource 4 Sodium dodecyl sulfate–polyacrylamide gel electrophoresis of the concentrated *Tc*-LysN culture supernatant (24 hr induction) that was incubated with trypsin for 60 minutes. Samples were withdrawn at indicated time points and mixed with reducing sample loading at heated for 5 minutes at 90°C before being loaded onto the gel. The "+" indicates samples that were incubated with trypsin and the "-" sign indicates samples that weren't incubated with trypsin. Lane M contains the marker proteins (PageRuler Prestained 10–180 kDa, Thermofisher).

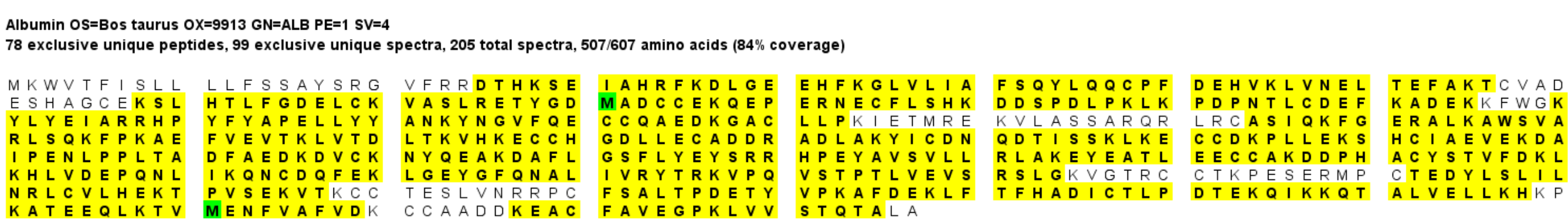

Online resource 5 Peptide sequence coverage of the in-solution digest of bovine serum albumin with *Tc*-LysN.
